# Taking advantage of a high-throughput flow cytometer for the implementation of an ADCC assay for regulatory compliance

**DOI:** 10.1016/j.btre.2020.e00456

**Published:** 2020-04-19

**Authors:** Rosa Camacho-Sandoval, Alexis Jiménez-Uribe, Alejandra V. Tenorio-Calvo, Carlos A. López-Morales, Leslie Muñoz-García, Alejandra Montes-Luna, Héctor Leonardo García-Xolalpa, Marco Velasco-Velázquez, Lenin Pavón, Sonia Mayra Pérez-Tapia, Emilio Medina-Rivero

**Affiliations:** a Unidad de Desarrollo e Investigación en Bioprocesos. Escuela Nacional de Ciencias Biológicas. Instituto Politécnico Nacional, Ciudad de México, Mexico; b Departamento de Farmacología y Unidad Periférica de Investigación en Biomedicina Translacional (CMN 20 de noviembre, ISSSTE), Facultad de Medicina, Universidad Nacional Autónoma de México, Ciudad de México, Mexico; c Laboratorio de Psicoinmunología. Dirección de Investigaciones en Neurociencias del Instituto Nacional de Psiquiatría, Ciudad de México, Mexico; d Sartorius de México S.A. de C.V. Tepotzotlán, Estado de México, Mexico

**Keywords:** ADCC, High-throughput flow cytometry, Validation bioassay

## Abstract

Technological advances allowed the development of high-throughput instruments such as IntelliCyt iQue Screener PLUS®. Here, we took advantage of this technology to transfer a previously validated cytotoxicity assay. The evaluated parameters were cell permeability, caspase activation and phosphatidyl serine exposure. The assay was accurate (r^2^ = 0.90), precise (%CV ≤ 18.90) and specific. These results showed that this technology is suitable to be used in control quality environments. In addition, the automation provided a faster acquisition and analysis of data with precise and accurate results. This application could be implemented to evaluate another *in vitro* mechanism of action of different biotherapeutics.

## Highlights

• High-throughput flow cytometry for the evaluation of ADCC potency.
• Optimization of biological assays for batch release testing of biopharmaceuticals.
• Quickly turn multi-parameter acquisition by flow cytometry.

## 1 Introduction

Flow cytometry (FC) is a widely adopted technology with several applications, mainly for the evaluation of cell properties (*i.e.* size and granularity), which are evaluated by detection of intrinsic or extrinsic fluorescence used to identify a large number of molecules. FC allowed the development of bioassays based on different mechanisms of action that result in cell proliferation, cytotoxicity, apoptosis and specific cytokine expression [1].

The main disadvantages of conventional flow cytometry are the significant delays related to saving individual sample associated files, and in the cell suspension sampling mechanism, which frequently involves time-consuming steps such as tube priming and flushing [2].

The evolution to high-throughput flow cytometry (HTFC) extends the capabilities of cell-based screening technologies. IntelliCyt iQue Screener PLUS® is a novel technological breakthrough that established the application of rapid flow cytometry by combining fast sample delivery with information-rich data on a cell-by-cell basis. This technology has several advantages, such as rate of analysis, small sample volumes and automated sample processing. This allows exploring applications in small molecule drug discovery and biotherapeutics. HTFC is used extensively in antibody screening, in order to select candidates with specific and optimized mechanism of action (MOA) and to detect structural modifications that could impact the biological activity [[3], [4], [5]].

Antibody-dependent cell-mediated cytotoxicity (ADCC) assays are essential to demonstrate primary *in vitro* studies for the clinical efficacy of many immunotherapies [6]. In a previous stage we developed and validated an ADCC assay to test the efficacy and potency of biopharmaceutical products, demonstrating cell death on target cells by membrane permeability using conventional flow cytometry [7]. Based on our experience in the development and validation of bioassays under GLP-cGMP environment, here we transferred our ADCC assay to a high-throughput technology, IntelliCyt iQue Screener PLUS®, by evaluating cell membrane permeability, caspase activation and phosphatidyl serine exposure as characteristics of death on target cells in the same sample with low volume of acquisition.

## 2 Materials and methods

### 2.1 Assay preparation

The preparation of the assay was based on the conditions we previously established for the ADCC assay using conventional flow cytometry [7]. In brief:

#### 2.1.1 Preparation of cells

Primary NK cells were isolated from peripheral blood from donors using negative selection by MACS® (Myltenyi Biotec, Bergisch Gladbach, Germany). We used Daudi Burkitt’s lymphoma cells (ATCC, Manassas, VA, USA) as target cells which were cultured in RPMI medium (GIBCO, NY. USA.) supplemented with fetal bovine serum (GIBCO, NY. USA.) and were harvested at 80 % of confluence. These samples were obtained from volunteers, who signed an informed consent according to an internal procedure.

#### 2.1.2 Formation of ADCC complex

Serial independent dilutions of rituximab (F. Hoffmann-La Roche Ltd. Basel, Switzerland) were prepared and incubated during 30 min along with the target cells (1 × 10^5^ cells/mL) in 96-well plates. Then, the effector cells were dispensed to the wells and cocultured for 4 h. The dose response curve was established 0.00013–1300 μg/mL with a 1:4 effector / target cells ratio.

### 2.2 Detection of cell death by flow cytometry

Cell death was determined by three different indicators: cell permeability, caspase activation and phosphatidyl serine exposure. Cell viability was determined as membrane integrity by inability to exclude a DNA binding dye due to compromises porous membrane using the FL3 detection channel. Caspase 3 and 7 activation was detected using the NucView® 488 Caspase-3/7 substrate, which upon cleavage by activated enzyme, results in a fluorescent signal detected by FL1 channel. Phosphatidyl serine exposure was detected by the binding of Annexin V to the cell surface detected by FL2 channel.

All the reagents we used to determine cell death were provided by Sartorius as part of the MultiCyt Apoptosis Kit (Sartorius, Göttingen, Germany). The IntelliCyt® iQue Screener PLUS® acquisition parameters used here were customized for the MultiCyt cell-based kit and preloaded to the instrument by the supplier.

### 2.3 Validation

The validation exercise focused on the assessment of: 4 PL fitting, specificity (against matrix), precision, and accuracy according to the ICH Q2 (R1) guideline and the USP <1033> Biological Assay Validation Chapter [8,9]. Statistical analyses were performed using the Graph Pad Prism 6.0 software (La Jolla, CA). All the experiments were performed by independent triplicates.

## 3 Results and discussion

In a previous report, we developed and validated an ADCC assay as per international guidelines, which could be implemented to evaluate different biopharmaceutical molecules bearing an Fc region [7].

Here we extrapolated our ADCC assay to a high-throughput flow cytometry system using the cell death characteristics of membrane permeability, caspase activation and phosphatidyl serine exposure as ADCC response on target cells.

**Fig. 1:**
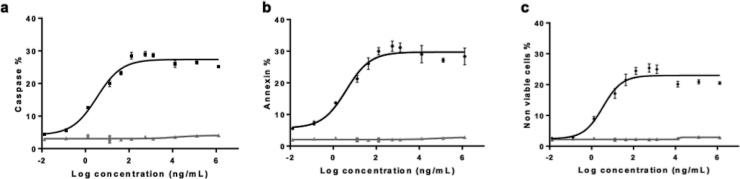
High-throughput flow cytometric analysis of death target cells by ADCC. Daudi cells were harvested and culture with rituximab and primary NK cells for ADCC assay as indicated in methodology. Cell death was determined by (a) caspase activation, (b) phosphatidyl serine exposure and (c) cell permeability. Black lines indicate the rituximab response at different concentrations while grey lines correspond to adalimumab (negative control). Error bars represent the standard deviation at each concentration level.

We observed a sigmoidal curve dependent on rituximab concentration with the three evaluated parameters. As shown in the Fig. 1 the dose-response curves fitted to the 4 PL model (r^2^ > 0.90) in all cases (Fig. 1a, b, c).

**Fig. 2:**
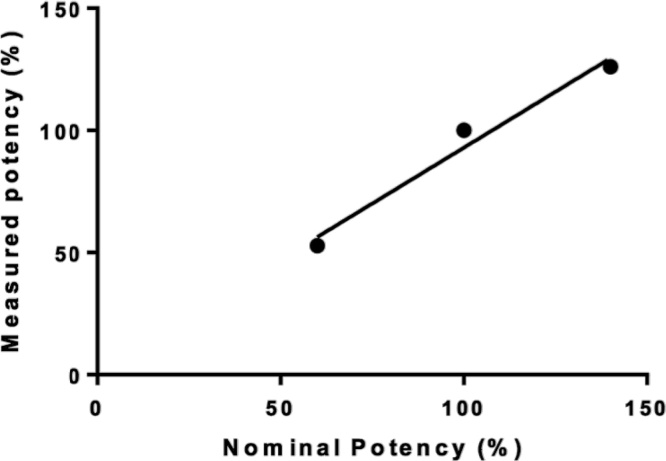
Accuracy of Annexin positive cells in response to rituximab. Accuracy was determined by dilutional linearity evaluated in a dilutional range of sample recoveries at 60, 100 and 140 %. Relationship between nominal and measured potency (EC50, r^2^ = 0.97, slope: 0.92).

The coefficients of variation at each concentration level were < 20 %. Specificity was confirmed by fitting the sigmoidal dose-response of adalimumab, as expected adalimumab recognized membrane-bound Tumor Necrosis Factor α (mTNF-α) expressed in the engineered CHO-K1 cells, and triggered the ADCC mechanism of action mediated by primary NK cells, unlike the negative control rituximab, which is targeted toward CD20, commonly expressed by B cells. Accuracy was determined by dilutional linearity evaluated in a dilutional range of sample recoveries at 60, 100 and 140 %. Among the three methods we used to determine cell death, annexin was selected to determine accuracy because exhibited the best linear behavior, which is desirable for assays intended to be used as routine tests for quality control purposes (Fig. 2). However, the other two determinations, cell permeability and caspase activation, could be also valuable to confirm the ADCC response making adjustments to achieve a suitable linear response. A summary of results is found in Table 1, we concluded that the three parameters demonstrated to be suitable for the assessment of ADCC.

**Table 1:** Measured validation parameters for ADCC induced by rituximab.

Characteristic	Parameter	Acceptance criteria	Results		
**4 PL model fitting**	Fitting to the 4 PL model^a^	r^2^ ≥ 0.90	Caspase	Annexin	Non-viable
			0.96^a^	0.90^a^	0.91^a^
**Specificity**	Fitting of the positive sample to the 4 PL model	r^2^ ≥ 0.90	r^2^ ≥ 0.90^a^		
	Negative sample do not fit 4 PL	Curve profile	Did not fit		
**Precision**	Repeatability^b^: Coefficient of variation (% CV) among independent triplicates at each concentration level of the dose-response curve	≤ 20.0 %	Caspase	Annexin	Non-viable
			1.4–13.6 %	2.1–18.9 %	3.5–18.3 %
**Accuracy**	Correspondence between the nominal potency and the measured potency obtained from dilutional linearity	r^2^ ≥ 0.90	0.97^c^		
	Slope of relative potency *vs* nominal potency	0.70 - 1.30	0.92^c^		
**System suitability**	Ratio between maximum response / minimum response of the positive sample	> 2.0	Caspase	Annexin	Non-viable
			6.5	5.2	9.5
	Fitting to the 4 PL model in a rituximab concentration range of the 1.3 × 10^−5^ -1.3 × 10^3^ μg/mL	r^2^ ≥ 0.90	r^2^ ≥ 0.90^a^		
	Repeatability	≤ 20.0 %	≤ 20.0 %^b^		

a This value summarizes the collective values from Fitting to the 4PL model.

b This value summarizes the collective repeatability values from Precision.

c Results of annexin.

The comparison of the results we obtained in a previous validated study using conventional FC detection [7] *versus* the HTFC results obtained here by evaluating the same attribute (cell permeability) and demonstrate that both methodologies complied with the pre-established acceptance criteria and are suitable for their intended use. As expected, automation of IntelliCyt iQue Screener PLUS® has the advantage of harnessing low sample volume more rapidly, thus decreasing variability in the acquisition and reducing the data analysis time. These advantages resulted in an increase in the number of tested samples without compromising the reliability of results.

## 4 Conclusions

The capabilities of the analytical platform of IntelliCyt iQue Screener PLUS® are suitable for the development of bioassays. In this study, we demonstrated that previous validated ADCC assay could be transferred to a high-throughput system that is also suitable for as an alternative for characterization and batch release purposes of biotherapeutic products such as rituximab under regulated environments. In this sense, there is a potential variety of applications that could be implemented to evaluate different MOA of biotherapeutics *in vitro*.

## Author statement

Camacho-Sandoval R and Jiménez-Uribe A, conducted all the assays, acquired and interpreted the data and results, and prepared a draft. Tenorio-Calvo A and López-Morales provided technical advisory, reviewed and corrected the manuscript. Muñoz-García L and Montes-Luna A, provided technical support in the execution of the assays. García-Xolalpa H provided technical advisory for the design of the assays, for the use of the IntelliCyt technology and also provided the reagents employed in this research. Velasco-Velázquez M and Pavón L provided a critical review to improve the manuscript. Pérez-Tapia S and Medina-Rivero E conceptualized this research and were in charge of the administration of the project.

## Declaration of Competing Interest

Hector Leonardo García-Xolalpa is employee of Sartorius de México S.A. de C.V. The other authors declare no conflict of interest.
